# Immune complexes can dampen inflammatory signaling at the mucosal surface during protective SIV vaccination

**DOI:** 10.1186/1742-4690-9-S2-O19

**Published:** 2012-09-13

**Authors:** AJ Smith, SW Wietgrefe, CS Reilly, PJ Southern, L Duan, KE Perkey, L Shang, R Johnson, AT Haase

**Affiliations:** 1 University of Minnesota , Minneapolis, MN, USA; 2 New England Primate Research Center, Southborough, MA, USA

## Background

The long-term goal for an efficacious HIV vaccine is to provide sterilizing protection from HIV infection. Thus far, this scenario has only been achieved experimentally using live-attenuated SIV vaccines. As such, great interest lies in identifying correlates of protection from a successful host response to pathogenic SIV. To this end, we have used a global genomics approach, tissue analysis, and explant cultures to identify immune complex (IC) signaling as an important component of a protective host response in the female reproductive tract (FRT) of animals vaccinated with the live-attenuated virus known as SIV _mac239_ ΔNef.

## Methods

RNA from cervical tissue was purified for microarray analysis. Significant genes were functionally classified and protein expression determined using single-cell analytical procedures for tissue sections. FRT tissue was removed from healthy, uninfected, adult Rhesus macaques and cervix isolated/dissected into small tissue pieces for ex vivo culturing. WT SIV_mac251_ 32H alone or SIV-specific ICs were added drop-wise to mucosal surfaces of explants and incubated for 24 hr at 37^o^C / 5% CO_2_.

## Results

A genome-wide transcriptomics analysis revealed selective enrichment of an anti-inflammatory program upon virus exposure in SIV_mac239_-ΔNef-vaccinated animals, with localized expression of these anti-inflammatory mediators in the mucosal epithelium of the FRT, coinciding with dampened inflammation, limited CD4^+^ T cell infiltration, and stunted virus replication. Explant cultures derived from the FRT of Rhesus macaques were used as a physiological platform to identify the inhibitory Fc receptor for IgG, FcγRIIB, and carbohydrates in the Fc portion of SIV-specific ICs as centrally important in mediating this anti-inflammatory signaling program in mucosal epithelial cells.

## Conclusion

These results highlight an unappreciated, non-neutralizing role for antiviral antibodies at mucosal surfaces and implicates the mucosal epithelial cell as an important host sensor that integrates external signals to elicit a host program that either promotes (inflammatory) or suppresses (anti-inflammatory) immunodeficiency virus infection.

